# MicroRNAome and Expression Profile of Developing Tooth Germ in Miniature Pigs

**DOI:** 10.1371/journal.pone.0052256

**Published:** 2012-12-18

**Authors:** Ang Li, Tieli Song, Fu Wang, Dayong Liu, Zhipeng Fan, Chunmei Zhang, Junqi He, Songlin Wang

**Affiliations:** 1 Molecular Laboratory for Gene Therapy and Tooth Regeneration, Beijing Key Laboratory of Tooth Regeneration and Function Reconstruction, Capital Medical University School of Stomatology, Beijing, China; 2 Department of Biochemistry and Molecular Biology, Capital Medical University School of Basic Medical Sciences, Beijing, China; Kyushu Institute of Technology, Japan

## Abstract

MicroRNAs (miRNAs) play important roles in the regulation of rodent tooth development, but little is known about their role in tooth development in large mammals. We identified 637 unique miRNA sequences in a large-scale screen for miRNA expression profiles in the developing lower deciduous molars of miniature pigs (*Sus scrofa*) using Illumina Solexa deep sequencing. These candidate miRNAs and another 105 known *Sus scrofa* miRNAs were included in the custom-designed microarray and used to analyze the miRNA expression profile in the bud, cap, early bell, and late bell stages of tooth development. Microarray analysis revealed 166 transcripts that were differentially expressed in the four stages. Bioinformatic analysis identified 18 key miRNAs, including let-7f, miR-128, miR-200b, and miR-200c, that might play key roles in tooth development. Taken together, our results not only identified the specific microRNAome and expression profile in developing lower deciduous molars of the miniature pig, but they also provided useful information for investigating the molecular mechanism of tooth development in the miniature pig.

## Introduction

Tooth development is controlled by genetic interactions involving growth factors, transcription factors, signal receptors, and diffusible morphogens [1]. Over 300 protein-coding genes have been identified during odontogenesis [2]. However, the underlying molecular pathway elements, such as the decisive secondary regulatory factors of the major genes responsible for controlling prenatal tooth growth, are poorly understood.

The discovery of microRNAs (miRNAs), tissue-specific and/or stage-specific expression, and their roles in cell biology have greatly expanded our knowledge regarding the regulation of gene expression [3]–[5]. Recent studies have revealed that miRNAs play important roles in the regulation of murine tooth development. For example, conditional knockout of Dicer1 (mature microRNA) in Pitx2-Cre mice and K14 transgenic mice result in significant aberrations in tooth shape and enamel formation [6], [7]. Using miRNA microarray analysis and RT-PCR, some researchers have found that miR-24, miR-31, miR-140, miR-141, miR-205, miR-200c, miR-875-5p, miR-455, miR-689, miR-711, and miR-720 may regulate tooth epithelial stem cell differentiation [6], [7]; others identified miR-133a, miR-200b, miR-206, and miR-218 as tooth-specific miRNAs, and that miR-141, miR-199b*, miR-200a, miR-200b, miR-200c, and miR-429 likely play a role in the renewal and differentiation of adult stem cells during stem cell-fueled incisor growth [8], [9]. However, mouse teeth differ from human teeth in both number and morphology [9].

As a large animal species, the pig is a suitable model organism for comparative genomics and biomedical studies [10]–[12]. In addition, the teeth and jaw bones of miniature pigs (minipigs) are similar to those of humans, as are the bite force of the molars and the hardness of the enamel [11]. Thus, minipigs are considered a suitable model for tooth development studies. In the present study, we used minipigs as a large animal model to investigate the miRNAs expression profiles of developing teeth.

## Results

### MicroRNAome of minipig tooth germ

**Figure 1:**
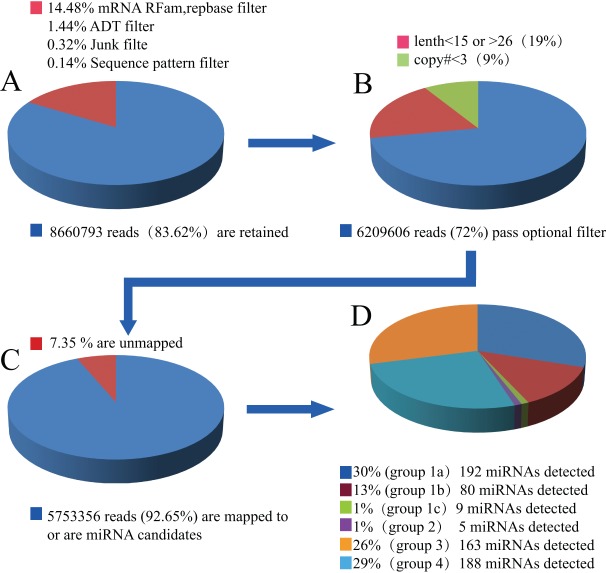
Analysis of sequencing data. To obtain mappable sequences from raw sequencing data, we used a series of digital filters to remove various unmappable sequencing reads. (A) Unique families of sequences were generated by sorting raw sequencing reads. “Impure” sequences were then removed by sample preparation, sequencing chemistry and processes, and the optical digital resolution of the sequencer detector. Unique sequences were pulled from selected databases, including mRNA, rRNA, tRNA, snRNA, snoRNA, and Repbase databases. (B) (*i*) A length filter was used to retain unique sequences of 16–26 nt. (*ii*) Sequences with copy numbers greater than the predefined cut-off number (default = 3) were also retained. (C) After masking the adaptor sequences and removing contaminated reads, the 92.65% mappable clean reads were processed for advanced analysis. (D) The unique mapped sequences were grouped as “unique sequences mapped to selected species pre-miRNAs in miRbase, and further mapped to pig genome and EST,” and divided into six groups.

In total, 10,356,944 reads (with redundancy) were obtained and sequenced from 12 samples of bud stage to late bell stage tooth germ of minipigs. Of these reads, 83.62% passed through the Adapter (ADT) dimmer, junk, mRNA, RFam, and Repbase filters (Figure 1A). To ensure credibility of the results, we retained high-copy sequences (counts ≥3). Based on the size distribution of all known miRNAs, 15–26 nt reads were selected as “clean reads” for further analysis (Figure 1B). Of these reads, the majority (91.75%) of the small RNAs were 21–23 nt in size, which is typical of small RNA Dicer-processed products (Figure S1).

The clean reads (sequences) were subjected to advance bioinformatics analysis (Figure S2) and divided into six groups: (1) Group 1a, 192 miRNAs corresponding to 127 known ssc (*Sus scrofa*) pre-miRNAs and miRNAs in the miRBase 15.0, which were also mapped to the pig genome or expressed sequence tags (EST). Specifically, 75 miRNAs were known but 117 were not previously identified porcine miRNA*s. (2) Group 1b, 80 miRNAs corresponding to 289 other known 22 mammalian species except for ssc pre-miRNAs and miRNAs in miRBase, which were further mapped to the pig genome and EST. Not all of the miRNAs were previously identified porcine miRNA*s. (3) Group 1c, nine miRNAs were mapped to the pig genome and EST (but different from the locations in gp1a and gp1b), and the extended sequences (60 nt) in the mapped genome positions were potential hairpin candidates. This kind of miRNAs was labeled PC (Predicted Candidate miRNAs) and previously unknown as porcine miRNAs/pre-miRNAs. (4) Group 2, five miRNAs corresponding to 24 other known 22 mammalian species pre-miRNAs and miRNAs in miRBase and the mapped pre-miRNAs were not further mapped to the pig genome, but the reads were mapped to the pig genome and were labeled PC. (5) Group 3, 163 miRNAs were correspond to 594 other known 22 mammalian species pre-miRNAs and miRNAs in miRBase and the mapped pre-miRNAs were not further mapped to the pig genome, and the reads were not mapped to pig genome either. This kind of miRNAs was labeled PN (Predicted Novel miRNAs) and previously unknown as porcine miRNAs/pre-miRNAs. (6) Group 4, 188 miRNAs corresponding to 370 candidate pre-miRNAs that were not mapped to 23 mammalian species pre-miRNAs in miRbase but predicted RNA hairpins derived from the pig genome, and the extended genome sequences form hairpins, which were labeled PC.

We identified a total of 637 unique miRNA sequences in the miRNA library of minipig tooth germ (Table S1); these unique sequences were identified from 8,424 individual sequences from 5,753,356 reads (Figure 1C, D). Among these sequences, 554 were not detected in miRbase 15. In addition, 188 small RNA sequences (Group 4) had no match in the selected mammalian miRBase, but fully matches with the pig genome that can fold into the characteristic stem-loop structure. Therefore, we concluded that these sequences belong to completely novel and pig-specific miRNAs. All 637 miRNA candidates were used in follow-up, custom-designed miRNA microarrays with 742 miRNA gene probes (including another105 known ssc miRNAs).

### MiRNA expression patterns during tooth development

Of the 616 detectable miRNA transcripts in the miRNA microarrays, 431 were expressed in E35, 358 in E45, 477 in E50, and 406 in E60 (Table S2). The differences in the numbers of miRNAs expressed in each embryonic stage were not significant (*p*>0.1, Figure S3). Pair-wise comparisons revealed that large numbers of miRNAs were differentially expressed between any two given stages: 37 between E35 and E45, 4 between E45 and E50, and 40 between E50 and E60. In addition, the greater the time interval, the more significant the difference in expression patterns; for example, 37 miRNAs were differentially expressed between E35 and E45, whereas 105 miRNAs were differentially expressed between E35 and E60 (Table S3). The similar expression levels and different patterns of miRNAs in different developmental stages of pig tooth germ support the importance and complexity of miRNAs in pig teeth morphology and functional development.

**Figure 2:**
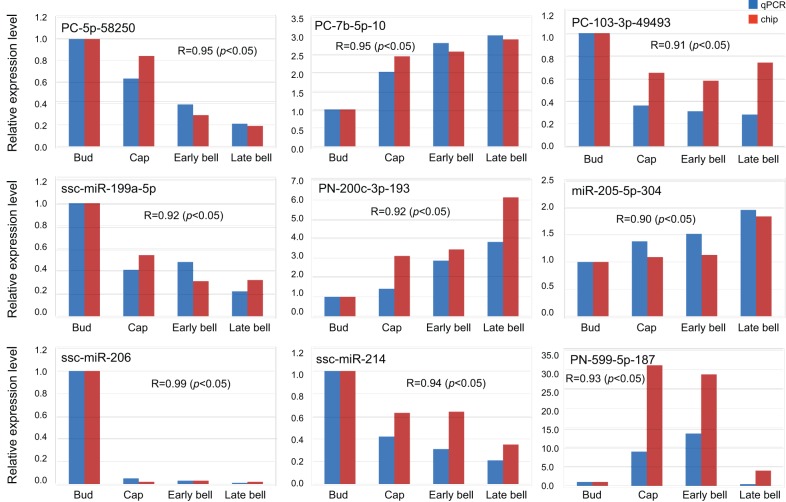
Validation of miRNAs by real-time RT-PCR. The expression levels of nine miRNAs were detected by real-time RT-PCR and microarray (chip). R represents the Pearson correlation coefficient. The expression levels of the nine miRNAs were in concordance with the normalized microarray data (R>0.9, *p*<0.05). Every time point was replicated three times using independently collected samples.

In order to validate the microarray results, nine representative miRNAs were chosen for real-time RT-PCR using 12 independent samples. The nine selected unique miRNAs were randomly selected in different expression patterns (i.e., expression levels reduced or increased through the four stages) based on multiple sample analysis of the miRNA microarray data (Table S4). The expression levels of all of the selected miRNAs were in concordance with the normalized microarray data (Pearson correlation coefficient R>0.9, *p*<0.05). In general, real-time RT-PCR validated the microarray analysis (Figure 2).

### Bioinformatic analysis of microarray data

**Figure 3:**
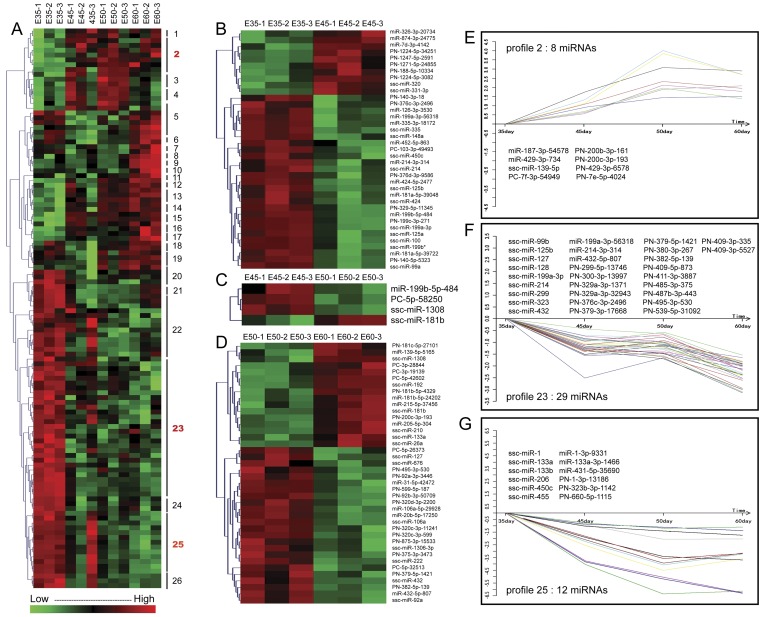
MiRNA expression profiles at different developmental stages by hierarchical clustering and STC analysis. (A) We found 116 miRNAs that were differentially expressed among the four developmental stages (*p*<0.05). Red indicates a gene that is highly expressed at that stage. Green indicates opposite gene that is lowly expressed at that stage. MiRNA expression was analyzed using the STC method, and 26 model profiles were defined. Three gene expression patterns were significant (*p*<0.05) (red boxes). (B–D) Pair-wise comparisons revealed that 37 miRNAs were differentially expressed (*p*<0.05) between E35 and E45, 4 between E45 and E50, and 40 between E50 and E60. (E–G) miRNA expression profiles 2, 23, and 25 in tooth germ tissue. The horizontal axis represents time, and the vertical axis shows the time series of miRNA expression after log-normalized transformation.

In hierarchical clustering analysis, 166 transcripts were differentially expressed in the four tooth developmental stages (*p*<0.1), 116 were significantly differentially expressed (*p*<0.05, Figure 3A), and 52 changed more significantly (*p*<0.01) including 31 with strong signals intensity (signal≥500, Table S4). Pair-wise comparisons revealed the important differentially expressed miRNAs between each developmental stage (Figure 3B–D). For example, between E35 and E45, miR-125 was the significantly differentially expressed miRNA (*p*<0.01), as the expression level was very high, this may be the key miRNAs in this stage. Between E45 and E50, PC-5p-58250 exhibited a greater change in expression, and it may be the key miRNA at this stage. Between E45 and E50, more differentially expressed miRNAs were observed; PN-92b-3p-50709 had the highest expression level and may be the most important miRNA at this stage (Table S4).

Using Series Test of Cluster (STC) analysis [13], we placed 116 differentially expressed miRNAs (*p*<0.05) into 26 possible expression pattern profiles (Figure S4). Three of that the profiles, which comprised 49 miRNAs, exhibited significantly different expression pattern among the four developmental stages (*p*<0.01, Figure 3E–G). Almost all of the 49 miRNAs had homologous isomers in humans and mice, which suggests that theses miRNAs are conservative and important in evolution, and that they may play important roles in teeth development. More interestingly, in addition to 31 known homologous miRNAs isomers in pig, another 18 unknown miRNAs were identified in pigs, all of which deserve further study (Table S5).

**Figure 4:**
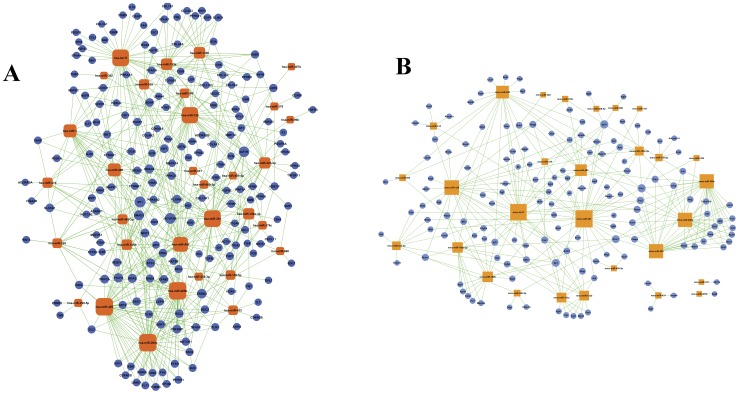
MicroRNA-gene network. Circular nodes represent genes, and rounded rectangle nodes represent miRNAs. The size of the nodes represents the power of the interrelation among the nodes, and edges between two nodes represent interactions between genes. The more edges a gene has, the more genes that interact with it, and the more central a role it has within the network. (A) The top five key miRNAs in the human network were hsa-miR-200b, hsa-miR-200c, hsa-miR-429, hsa-miR-381, and hsa-let-7f. The top five key mRNAs were SP1, ACVR2B, IGF1R, SMAD2, and ERBB4. (B) The top five key miRNAs in the mouse network were mmu-miR-381, mmu-let-7f, mmu-miR-128, mmu-miR-200b, and mmu-miR-200c. The top five key mRNAs were Ets1, Igf1r, Nr3c1, Sp1, and Frs2.

Subsequent analysis to detect the possible target genes and functions of 49 miRNAs identified 1,841 predicted miRNA-mRNA interaction sites from the pig database, 7,717 from the mouse database (including 1,893 genes related to tooth development), and 11,761 from the human database (including 3,814 genes related to tooth development). By referring to known interactions between miRNAs and genes in mice and humans, we created a diagram of a miRNA gene regulatory network (Figure 4A, B) that quantitatively separates the core regulatory functions of miRNAs and their target genes. The high degree of connectivity between the miRNA-mRNA pairs suggests that these miRNAs play critical roles during tooth development.

Using the diagram of the miRNA-gene regulatory network, we found that 12 miRNAs of the 38 candidates have no target gene in the mouse or human gene annotation. Comprehensive consideration of the signal intensity (signal≥500) (Table S6) let to the prediction of 18 key miRNAs, including let-7f, miR-128, miR-200b, and miR-200c (Table 1). These miRNAs provide a valuable candidate list of tooth growth and development-related miRNAs.

**Table 1:** Key miRNA in tooth germ.

No	miRNA	probe_id	related signaling pathway in pig	miRNA function in tooth development
**1**	let-7f	PC-7f-3p-54949	TGF-beta	unknown but highly expressed in mouse incisor epithelium and mesenchyme [6]
**2**	miR-99b	ssc-miR-99b	others	unknown
**3**	miR-125b	ssc-miR-125b	TGF-beta	unknown but highly expressed in mouse incisor epithelium and mesenchyme [6]
**4**	miR-128	ssc-miR-128	Wnt	unknown
**5**	miR-133a	ssc-miR-133a	others	unknown but expressed in mouse tooth germ
**6**	miR-133b	ssc-miR-133b	others	unknown
**7**	miR-199a-3p	ssc-miR-199a-3p	Wnt	unknown but highly expressed in mouse incisor mesenchyme [6], [9]
**8**	miR-200b	PN-200b-3p-161	TGF-beta	unknown but expressed in mouse incisor labial cervical loop epithelium [8], [9]
**9**	miR-200c	PN-200c-3p-193	others	unknown but highly expressed in mouse incisor and labial cervical loop epithelium [8], [9]
**10**	miR-206	ssc-miR-206	TGF-beta	unknown
**11**	miR-214	ssc-miR-214	Wnt	indirect affects amelogenesis [26]
**12**	miR-379	PN-379-5p-1421	others	unknown
**13**	miR-380	PN-380-3p-267	others	unknown
**14**	miR-382	PN-382-5p-139	Wnt	unknown
**15**	miR-409	PN-409-3p-335	Wnt	unknown
**16**	miR-455	ssc-miR-455	others	unknown but prevalent at the mouse tooth germ cytodifferentiation stage [7], [8]
**17**	miR-487b	PN-487b-3p-443	others	unknown
**18**	miR-495	PN-495-3p-530	others	unknown

## Discussion

The *Sus scrofa* genome is approximately 2.7 billion base pairs in size and is phylogenetically closer to the human genome than the genomes of rodent species. However, the miRBase 15.0 database (April 2010) only reports 188 mature miRNAs in ssc, much fewer than the number of miRNAs identified in other species (e.g., 940 mature miRNAs in human and 590 mature miRNAs in mice). Several studies have report new, unique swine miRNAs [14]–[18], but no report is yet available on miRNA expression profiles in the teeth of pigs. Currently, extracting the small RNA from the corresponding organization, high-throughput sequencing, and using biological informatics to identify new miRNAs is the most rapid and accurate method. Using Illumina Solexa deep sequencing, we identified 637 unique miRNAs in the developing lower deciduous molars of minipigs. Li et al [19] revealed 623 pre-miRNAs that encode 771 unique miRNAs in porcine mixture tissues over the entire lifetime of the pig. These numbers are very close to our results and indicate that miRNAs are expressed continuously during tooth development.

We designed a specific custom miRNA microarray chip to analyze the miRNAs expression profiles in the bud, cap, early bell, and late bell stages of tooth development [20]–[22]. Microarray analysis revealed 166 transcripts that were differentially expressed in the four stages. Next, we preliminarily determined 49 differentially expressed miRNAs using STC analysis. In additional screening, we used a well-established miRNA-target dataset generated by TargetScan to investigate the possible function of these miRNAs with the aid of gene function annotation methods, include Gene Ontology (GO) and Kyoto Encyclopedia of Genes and Genomes (KEGG) progress. Because functional genetic information from the pig is insufficient, we chose homologous rat and human miRNAs (Table S5) for target gene annotation. The target sites were characterized as evolutionarily conserved in three species (human, mouse, and pig), a criterion that acted as a good filter for false positive assignments of miRNAs to genes [23]. Finally, we integrated the information through miRNA-mRNA network analysis (Figure 4) and determined the 26 miRNAs relevant to mouse and human GO/Pathway target genes that intersect (degree≥1) at the same time in the 38 candidate miRNAs (Table S6). Removing 8 miRNAs with strength less than 500, we finally obtained 18 miRNAs key miRNAs profiles (Table 1).

The TGF-beta and the Wnt signaling pathway are known to play an essential role in the activation of odontogenic mesenchyme during early tooth development [24], [25]. Based on the KEGG data from pigs, humans and mice, we built a pathway interaction network (*p*<0.05, Table S7), which includes the TGF-beta and Wnt signaling pathway and will provide useful clues regarding how miRNAs influence tooth development through these pathways.

Among the 18 predicted key miRNAs (Table 1), some may be associated with porcine TGF-beta and Wnt signaling, but the function of these miRNAs in tooth development remains unknown. Seven key miRNAs including let-7f, miR-125b, miR-133a, miR-199a, miR-200b, miR-200c and miR-455 are highly expressed in the mesenchyme or epithelium of different tooth development stages, but their functions have not been elucidated [6], [8]. MiR-200b and miR-200c are both important regulatory miRNAs, but only miR-200b regulates the porcine TGF-beta signaling pathway. MiR-214 is the most highly expressed miRNA, and the most likely “tooth-specific” miRNA, but recent studies of the role of miR-214 in tooth germs suggest that miR-214 indirectly affects amelogenesis [26].

In general, this study presents the most complete microRNAome and transcriptome profiles for evaluating miRNAs abundance in teeth at specific time points during fetal development in minipigs. Identification of these key miRNAs though bioinformatics analysis provides an initial group of expressed miRNAs that change in abundance during specific developmental stages and, therefore, may target genes that regulate this process. These results are a prelude to advancements in pig tooth biology, as well as the use of pigs as model organism for human tooth growth and development studies.

## Materials and Methods

### Ethics Statement

The Wuzhishan minipigs used in this experiment were obtained from the Kexing Laboratory Animal Company of Beijing, China. Experiments were performed according to the Regulations for the Administration of Affairs Concerning Experimental Animals (Ministry of Science and Technology, China, revised in June 2004), and approved by the Animal Care and Use Committees of Capital Medical University, Beijing, China under permit No.CMU-B20100106. The animals were allowed access to feed and water ad libitum under normal conditions and humanely sacrificed as necessary to ameliorate suffering. In brief, pregnant sows were anesthetized with a combination of 6 mg/kg ketamine chloride and 0.6 mg/kg xylazine, and pregnancy and the fetal state roughly determined by B-mode ultrasonography. After removing the fetuses by cesarean section, the pregnant sows were sacrificed by over-anesthetization.

### Sample Preparation

Twelve pregnant sows were humanely sacrificed at four prenatal stages (three pigs per time point): embryonic day 35 (E35), E45, E50, and E60, which cover the major morphological and physiological changes of the deciduous molar tooth including the bud, cap, early bell, and late bell stages [27], [28] of pig tooth germ growth and development throughout pregnancy. After surgically removing the fetuses, germ tissue samples from deciduous molar tooth were removed from the mandibles under a microscope. The samples were immediately frozen in liquid nitrogen and stored separately at −80°C until used for analysis (Table S8).

### Solexa Sequencing

Total RNA was isolated using an RNA purification kit (Illumina, San Diego, USA) according to the manufacturer's instructions. Equal quantities (10 μg) of small RNA isolated from 12 individual pigs were pooled. Approximately 120 μg of small RNA representing each stage of development was used for library preparation and sequencing. The libraries were sequenced using Genome Analyzer GAIIx (Illumina) according to the vendor's recommended protocol for small RNA sequencing by sequencing-by-synthesis technology.

### Data Processing

The image files generated by the sequencer were processed to produce digital-quality data. After masking the adaptor sequences and removing contaminated reads with Illumina's Pipeline v1.5 software, clean sequences were processed for computational analysis using the proprietary software package ACGT101-miR v3.5 (LC Sciences, Houston, USA) [19]. Various “mappings” were performed with unique sequences against mammalian pre-miRNA and mature miRNA sequences contained in miRBbase 15.0. Mappings were also done with hairpin-precursor miRNAs of interest against the ssc genome from the Sanger Sscrofa9.53 database (ftp://ftp.sanger.ac.uk/pub/S_scrofa/assemblies/Ensembl_Sscrofa9/) and EST depository (ftp://ftp.ncbi.nih.gov/repository/dbEST) (Figure S2).

### Custom-designed MiRNA Microarray

A total 637 newly determined, unique miRNA sequences and 105 known *Sus scrofa* miRNAs were included for custom-designed microarrays. We finally confirmed 742 miRNA gene probes using custom-designed miRNA microarrays (Table S9). To ensure stability, every probe was performed in quadruplicate. Next, we carried out a comparative miRNA expression profile analysis across tooth germ samples independently collected from pig embryos on E35, E45, E50, and E60.

The microarray expression assay was performed by a service provider (LC Sciences) using a similar approach as Teferedegne et al. [29]. Briefly, total RNA (5 μg) from each developmental stage was size fractionated (<300 nt) using a YM-100 microcon centrifugal filter (Millipore, Billerica, MA). An oligonucleotide tag was ligated to the poly (A) tail for later fluorescent-dye staining. Hybridization was performed overnight on a μParaflo microfluidic chip using a micro-circulation pump (Atactic Technologies, Houston, TX). The detection probes were made by in situ synthesis using photogenerated reagent (PGR) chemistry. After RNA hybridization, tag-conjugating Cy3 and Cy5 dyes were circulated through the microfluidic chip. Fluorescence images were collected using a laser scanner (GenePix 4000B, Silicon Valley, CA) and digitized using Array-Pro image analysis software (Media Cybernetics, Bethesda, MD). Pairs of labeled samples from different stages were hybridized to dual channel microarrays. LC Sciences performed the miRNA microarray analyses, and the details of the multiple sample analysis can be found in Text S1. The microarray data comply with Minimum Information About a Microarray Experiment (MIAME) requirements and have been deposited in the Gene Expression Omnibus data base (accession number GPL15885).

### Quantitative real-time RT-PCR

To validate the microarray data, we used a previously described miRNA quantification method [30]. RNA samples used in qPCR validation experiments were isolated from 12 different pregnant pigs used in the array assay. DNAStar version 6.1.3 software was used to design the primer sequences (Table S10). First, 100 ng of total RNA was reverse transcribed using 100 U of M-MLV reverse transcriptase (Takara: D2639A) and 1 uM stem-loop RT primer in a 7900 Thermocycler (Applied Biosystems, Carlsbad, USA) with incubation at 42°C for 60 min and 70°C for 15 min. The samples were then held at 4°C. Real-time PCR was performed using Platinum SYBR Green qPCR SuperMix-UDG (Invitrogen: 11733-038). Porcine ssc-miR-24 [31] was used as an internal control, and all reactions were run in triplicate. The ΔΔCT method was used to determine the differences in expression between surveyed stages [32].

The expression levels in each developmental stage were compared to the bud stage, so that the raw real-time PCR data could be scaled to the fold change data (microarray data also use the same method). The concordance between real-time PCR and microarray was statistically analyzed by Pearson's correlation coefficient according to the fold change data. Significance was set to *p*<0.05 and R>0.9.

### Bioinformatics Analysis

STC analysis (Genminix Informatics Ltd, Shanghai, China) was performed for the differentially expressed miRNAs [13]. Unique profiles were defined and significant profiles selected (*p*<0.01). For miRNAs expressed in significant amounts, we used the miRanda algorithm base (http://www.microrna.org/microrna/home.do) to predict miRNA target genes in the pig and TargetScan (http://www.targetscan.org/) for the mouse and human. GO terms (http://www.geneontology.org/) and KEGG Pathway (http://www.genome.jp/kegg/) annotation of the miRNA targets were found using the DAVID (The Database for Annotation, Visualization and Integrated Discovery) gene annotation tool (http://david.abcc.ncifcrf.gov/). We chose only GOs terms with *p*<0.00001 and FDR <0.00001, and Pathways with *p*<0.05 and FDR <0.05.

Through target genes function (GO) and Pathway-significant analysis, we obtained significant GO/Pathway target genes. Next, we took the intersection genes belonging to GO and the Pathway at same time. According to the attributes of the intersecting target genes and miRNAs, the MicroRNA-Gene-Network, representing the critical miRNAs and their targets, was established according to the miRNA degree (i.e., the contribution of one miRNA to the genes around or the contribution one gene to the miRNAs around it). The key miRNA and gene in the network always have the largest degrees [33]. Additional details of the methodology can be found in Text S1.

## Supporting Information

Figure S1 **Length distribution of mappable data.** The majority of the small RNAs were 21–23 nt in size, and they accounted for 91.75% of the total mappable reads.
(EPS)

Figure S2 **Advanced biological information analysis workflow.** First, the clean sequences were BLASTed against known porcine pre-miRNAs and other mammals pre-miRNAs from miRBase 15.0. The 23 mammalians species included were cfa (*Canis familiaris*), eca (*Equus caballus*), mdo (*Monodelphis domestica*), age (*Ateles geoffroyi*), lla (*Lagothrix lagotricha*), sla (*Saguinus labiatus*), mmu (*Macaca mulatta*), mne (*Macaca nemestrina*), pbi (*Pygathrix bieti*), ggo (*Gorilla gorilla*), has (*Homo sapiens*), ppa (*Pan paniscus*), ptr (*Pan troglodytes*), ppy (*Pongo pygmaeus*), ssy (*phalangus syndactylus*), lca (*Lemur catta*), oan (*Ornithorhynchus anatinus*), cgr (*Cricetulus griseus*), mmu (*Mus musculus*), rno (*Rattus norvegicus*), bta (*Bos taurus*), oar (*Ovis aries*), and ssc (*Sus scrofa*). The mapped pre-miRNAs were then BLASTed against the *S. scrofa* genome to determine their genomic and locations. The unmapped sequences were BLASTed against the *S. scrofa* genome and hairpin RNA structures predicated from the adjacent 60 nt sequences in both directions, in accordance with criteria generated using UNAfold software for mammalian pre-miRNAs in the miRBbase 15.0. The mature miRNA transcripts identified from the mapped sequences were divided into six groups.
(EPS)

Figure S3 **MiRNA expression number in each group.** Of the detectable miRNAs transcripts, 373–468 were expressed in E35, 305–459 in E45, 418–511 in E50, and 331–451 in E60. However, the differences in the number of miRNAs expressed at each stage were not significant (*p* = 0.240).
(EPS)

Figure S4 **MiRNA expression patterns yielded by STC analysis.** The expression patterns of 116 microRNAs were analyzed and 26 model profiles generated using the STC method. Each box represents a model expression profile. The upper number in the profile box is the model profile number, and the lower number is the p value. Three gene expression patterns were significant (*p*<0.01) (red boxes).
(EPS)

Table S1 Porcine tooth germ miRNA candidates revealed by Solexa deep sequencing.
(XLS)

Table S2 Detectable transcripts in each sample of different tooth germ development stages.
(XLS)

Table S3 Summary of differentially expressed miRNAs.
(XLS)

Table S4 Differentially expressed miRNAs detected by hierarchical clustering analysis of the microarrays data.
(XLS)

Table S5 Significant miRNA expression patterns.
(XLS)

Table S6 The function of mainstream trend miRNA in different species.
(XLS)

Table S7 The Pathway-Network list.
(XLS)

Table S8 Sample sets in this study.
(XLS)

Table S9 Probe list for microarray analysis.
(XLS)

Table S10 Primer sequences for real-time PCR.
(XLS)

Text S1 **Supplementary methods weren't discussed directly in the text.**
(DOC)
