# Supplementary material for: MicroRNAome and Expression Profile of Developing Tooth Germ in Miniature Pigs
Source: PLoS One. 2012 Dec 18;7(12):e52256. doi: 10.1371/journal.pone.0052256 (PMC3525553; doi:10.1371/journal.pone.0052256)
Supplement: Text S1 — Supplementary methods weren't discussed directly in the text. (DOC) [file pone.0052256.s015.doc]

**Multiple sample analysis of the miRNAs microarray data**

**Normalization:** Data were analyzed by first subtracting the background and then normalized using a cyclic LOWESS filter (locally-weighted regression) [1]. The normalization is to remove system related variations, such as sample amount variations, different labeling dyes, and signal gain differences of scanners so that biological variations can be faithfully revealed.

**Data adjustment:** Data adjustment includes data filtering, Log2 transformation, and gene centering and normalization. The data filtering removes miRNAs with (normalized) intensity values below a threshold value of 32 across all samples. The Log2 transformation converts intensity values into Log2 scale. MiRNAs centering and normalization transform the Log2 values using the mean and the standard deviation of individual miRNAs across all samples. A transcript to be listed as detectable must meets at least two conditions: signal intensity higher than 3 × (background standard deviation) and spot CV < 0.5. CV is calculated by (standard deviation)/(signal intensity). When repeating probes were present on an array, a transcript was listed as detectable only if the signals from at least 50% of the repeating probes were above detection level.

**t-Test:** t-Test is performed between “control” and “test” sample groups[2]. T-values are calculated for each miRNAs, and p-values are computed from the theoretical t-distribution. miRNAs with p-values below a critical p-value (typically 0.01 or 0.05) are selected for cluster analysis. All above data processes are carried out using in-house developed computer programs.

**Clustering:** The clustering is done using hierarchical method and is performed with average linkage and euclidean distance metric [3]. The clustering plot is generated using TIGR MeV (Multiple Experimental Viewer) software from The Institute for Genomic Research.

**A Series Test of Cluster (****STC)** **analysis**

STC is a kind of hierarchical cluster methods to analysis the trend of expression which can precisely and directly screen out the most significant and mainstream influence miRNAs with the sample order change. We selected differential expression genes at a logical sequence. In accordance with different signal density change tendency of genes under different situations, we identify a set of unique model expression tendencies. The raw expression values were converted into log_2_ ratio. Using a strategy for clustering short time-series gene expression data, we defined some unique profiles. The expression model profiles are related to the actual or the expected number of genes assigned to each model profile. Significant profiles have higher probability than expected by Fisher’s exact test and multiple comparison test [4,5]. *P*-Value represents the significant level compared between the actual miRNAs quantity and random distribution theory miRNAs number; the smaller the *P*-Value, the more notable of the influence the miRNAs expression trend change by biological samples.

**GO terms and KEGG pathway** **annotation of the miRNA targets**

GO analysis was applied to analyze the main function of the differential expression genes according to the Gene Ontology which is the key functional classification of NCBI [6]. Generally, Fisher’s exact test and $\chi^{2}$test were used to classify the GO category, and the false discovery rate (FDR) (Dupuy D et al. 2007) was calculated to correct the P-value，the smaller the FDR, the small the error in judging the p-value. The FDR was defined as $FDR=1-\frac{N_{k}}{T}$, where $N_{k}$ refers to the number of Fisher’s test *P*-values less than$\chi^{2}$test *P*-values. We computed *P*-values for the GOs of all the differential genes. Enrichment provides a measure of the significance of the function: as the enrichment increases, the corresponding function is more specific, which helps us to find those GOs with more concrete function description in the experiment. Within the significant category, the enrichment Re was given by:$\mathrm{Re}=(n_{f}/n)/(N_{f}/N)$where $n_{f}$ is the number of differential genes within the particular category, $n$is the total number of genes within the same category, $N_{f}$is the number of differential genes in the entire microarray, and $N$is the total number of genes in the microarray[7].

Similarly, Pathway analysis was used to find out the significant pathway of the differential genes according to KEGG, Biocarta and Reatome. Still, we turn to the Fisher’s exact test and $\chi^{2}$test to select the significant pathway, and the threshold of significance was defined by *P*-value and FDR. The enrichment Re was calculated like the equation above[8,9].

**MicroRNA- gene- network**

The relationship of the MicroRNA and genes were counted by their differential expression values, and according to the interactions of MicroRNA and genes in Sanger MicroRNA database to build the MicroRNA-Gene-Network. The adjacency matrix of MicroRNA and genes A=[a_i,j_] is made by the attribute relationships among genes and MicroRNA, and a_i,j_ represents the relation weigh of gene i and MicroRNA j. In the MicroRNA-Gene-Network, the circle represents gene and the shape of square represents MicroRNA, and their relationship was represented by one edge. The center of the network was represents by degree. [10,11].

**References:**

1. Bolstad BM, Irizarry RA, Astrand M, Speed TP (2003) A comparison of normalization methods for high density oligonucleotide array data based on variance and bias. Bioinformatics 19: 185-193.

2. Pan W (2002) A comparative review of statistical methods for discovering differentially expressed genes in replicated microarray experiments. Bioinformatics 18: 546-554.

3. Eisen MB, Spellman PT, Brown PO, Botstein D (1998) Cluster analysis and display of genome-wide expression patterns. Proc Natl Acad Sci U S A 95: 14863-14868.

4. Ramoni MF, Sebastiani P, Kohane IS (2002) Cluster analysis of gene expression dynamics. Proc Natl Acad Sci U S A 99: 9121-9126.

5. Miller LD, Long PM, Wong L, Mukherjee S, McShane LM, et al. (2002) Optimal gene expression analysis by microarrays. Cancer Cell 2: 353-361.

6. Ashburner M, Ball CA, Blake JA, Botstein D, Butler H, et al. (2000) Gene ontology: tool for the unification of biology. The Gene Ontology Consortium. Nat Genet 25: 25-29.

7. Schlitt T, Palin K, Rung J, Dietmann S, Lappe M, et al. (2003) From gene networks to gene function. Genome Res 13: 2568-2576.

8. Kanehisa M, Goto S, Kawashima S, Okuno Y, Hattori M (2004) The KEGG resource for deciphering the genome. Nucleic Acids Res 32: D277-D280.

9. Draghici S, Khatri P, Tarca AL, Amin K, Done A, et al. (2007) A systems biology approach for pathway level analysis. Genome Res 17: 1537-1545.

10. Joung JG, Hwang KB, Nam JW, Kim SJ, Zhang BT (2007) Discovery of microRNA-mRNA modules via population-based probabilistic learning. Bioinformatics 23: 1141-1147.

11. Shalgi R, Lieber D, Oren M, Pilpel Y (2007) Global and local architecture of the mammalian microRNA-transcription factor regulatory network. PLoS Comput Biol 3: e131.
